# An updated review on animal models to study attention-deficit hyperactivity disorder

**DOI:** 10.1038/s41398-024-02893-0

**Published:** 2024-04-11

**Authors:** Daegeon Kim, Dhananjay Yadav, Minseok Song

**Affiliations:** Department of Life Science, Yeungnam University, Gyeongsan-si, South Korea

**Keywords:** Neuroscience, Human behaviour

## Abstract

Attention-deficit hyperactivity disorder (ADHD) is a neuropsychiatric disorder affecting both children and adolescents. Individuals with ADHD experience heterogeneous problems, such as difficulty in attention, behavioral hyperactivity, and impulsivity. Recent studies have shown that complex genetic factors play a role in attention-deficit hyperactivity disorders. Animal models with clear hereditary traits are crucial for studying the molecular, biological, and brain circuit mechanisms underlying ADHD. Owing to their well-managed genetic origins and the relative simplicity with which the function of neuronal circuits is clearly established, models of mice can help learn the mechanisms involved in ADHD. Therefore, in this review, we highlighting the important genetic animal models that can be used to study ADHD.

## Introduction

Attention-deficit hyperactivity disorder (ADHD) is the most common neurobehavioral disorder in childhood and is characterized by inattention, impulsivity, and hyperactivity. Although ADHD is thought to be a crippling and frequent illness that only arises in infancy, recent studies indicate that it persists in adulthood in 30–70% of patients [1, 2]. Furthermore, poor cognitive impulsiveness, forgetfulness, planning deficits, poor time management, and impulsive conduct are prevalent in children with ADHD. Adults are diagnosed with ADHD by examining clinical abnormalities, such as: hyperactive-impulsive (ADHD-HI), predominantly inattentive (ADHD-PI), or combination (ADHD-C) subtypes.

ADHD is one of the most frequent juvenile disorders, with a prevalence of 3–5%. About half of the children affected by ADHD continue to experience symptoms as adults [3]. Numerous ADHD symptoms, such as-hyperactivity/impulsivity and attention impairment- must appear before the age of 12 years.

**Fig. 1:**
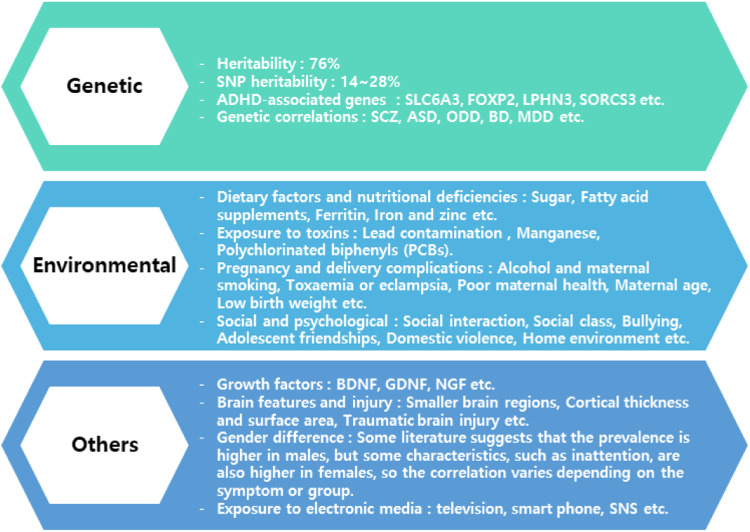
The common risk factor of ADHD. SNP single-nucleotide polymorphism, SLC6A3 solute carrier family 6 member 3, FOXP2 Forkhead box P2, LPHN3 Latrophilin-3, SORCS3 Sortilin-related VPS10 domain containing receptor 3, SCZ schizophrenia, ASD autism spectrum disorder; Odd oppositional defiant disorder, BP bipolar disorder, MDD major depressive disorder, BDNF brain-derived neurotrophic factor, GDNF glial cell line-derived neurotrophic factor, NGF nerve growth factor.

ADHD has been demonstrated to be comorbid with a number of different mental diseases in addition to this primary symptomatology. Mood, anxiety, oppositional defiance, and conduct disorders are the most frequent among children [4]; whereas in adults, different comorbidities occur, such as: major depressive disorder, social phobia, and substance abuse [5]. Emotional lability or dysregulation plays an underlying role in the development of ADHD symptoms [6]. In literature, there are various risk factors that play in elevating the proportion of ADHD patients including genetic [7, 8], environmental factors [9–11] and other related factors [12–14] that are briefly discussed and highlighted by various researcher obtained from different studies (Fig. 1).

Seo et al. [15] used national representative data collected between 2008 and 2018 to investigate the prevalence and comorbidities of ADHD among adults, children, and adolescents in Korea. They reported that ADHD prevalence rates for children/adolescents had increased steeply over that decade, from 127.1/100,000 in 2008 to 192.9/100,000 in 2018, increasing 1.47 and 10.1 times in children/adolescents (≤18 years) and adults (>18 years), respectively. According to the study, a significant proportion of ADHD patients in Korea are either misdiagnosed or undertreated.

The cause of ADHD remains unknown, but mounting evidence points to a hereditary component to its occurrence. Now with the help of recently developed genetic models, we may be able to comprehend the behavior of animals manifested by the presence of an attention-deficit disorder, hyperactivity, impulsivity, or all three traits in a single animal. Several animal models of ADHD have been proposed; however, genetically modified animals are the most promising models for displaying ADHD symptoms. ADHD models differ in terms of pathophysiological abnormalities and the capacity to imitate behavioral symptoms and predict pharmaceutical responses. Their varied nature could be attributed to the lack of sufficient knowledge on ADHD biology from clinical data based on human studies, which is why researchers are unable to determine which model best mimics ADHD or other subtypes. As per the recent research on ADHD, the models used should be classified as animal models of symptoms similar to ADHD rather than exact models of ADHD [16].

In this review, we discuss the most notable animal models that could be valuable for studying ADHD with a particular focus on genetic models. Various models include: dopamine transporter (DAT) knockout mice, spontaneously hypertensive rats (SHR), steroid sulfatase, coloboma mice, and alpha-synuclein-lacking mice.

### Animal model and criteria for good animal models

McKinney (1988) stated the requirement of animal models for “experimental preparations developed in one species for the purpose of studying phenomena occurring in another species [17, 18]”. This definition is still valid for clinical researchers. Certain criteria from animal models- such as: etiology, genetic resemblance, physiological processes, and treatment-can be used to study human psychological disorders. Three forms of validity were chosen: predictive, face, and construct. The predictive validity of a model is determined by whether it properly selects a pharmacological treatment with equivalent clinical potency without omission or commission errors. Face validity is determined by how closely a model mimics illnesses in different ways. Construct validity is evaluated by whether both the model’s behavior and the features of the disorder traits can be unambiguously interpreted and are homologous, and whether the features being modeled have a well-established empirical and theoretical relationship with the disorder [19].

### Dopamine transporters knock out mice

DAT is expressed in all dopamine (DA) neurons but is known to reuptake extracellular DA in the synaptic cleft of the dopamine system [20–22]. Therefore, dopamine transporter knockout (DAT-KO) using various methods causes an increase in DA by reducing extracellular DA clearance [22]; hence, the level of extracellular DA can be increased by nearly five times [23].

**Fig. 2:**
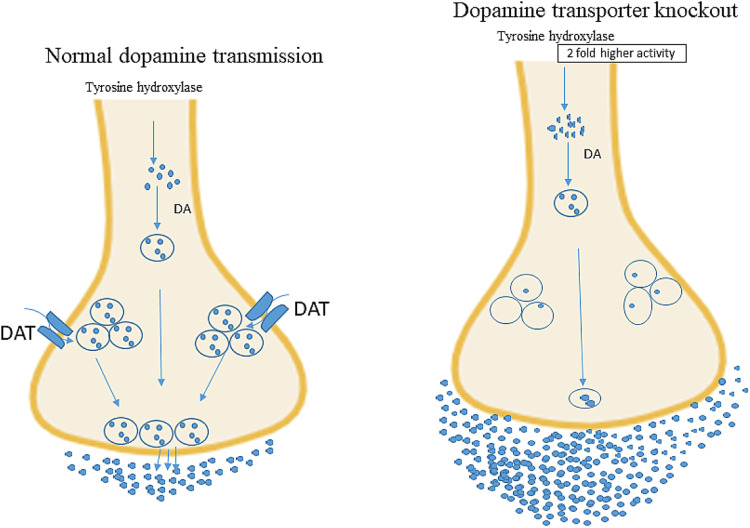
Differences in dopamine transmission in normal and dopamine transporter knock out (DAT-KO). Figure was modified and redrawn from Efimova et al. [37].

Figure 2 shows the dopamine homeostasis in normal and DAT-KO mice. The right picture depicts DAT-KO mice, where the synthesis of DA is double as compared to that in normal mice, and the neuronal DA concentration is drastically lower, whereas extracellular DA is increased five-fold. DAT-KO mice lacked auto-receptor function.

DA is thought to play an important role in ADHD; however, other neurotransmitters are also involved. Transgenic mice have become indispensable tools for analyzing the role of genetic factors in the pathogenesis of human diseases. Although rodent models cannot fully recapitulate complex human psychiatric disorders such as ADHD, transgenic mice offer an opportunity to directly investigate the specific roles of novel candidate genes identified in patients with ADHD in vivo. Targeting genes implicated in DA transmission, such as the gene encoding the dopamine transporter (DAT1), has led to the development of several knockout and transgenic mouse models proposed as ADHD models. These mutant animals provide researchers with the opportunity to assess the role of dopamine-related processes in brain diseases, analyze the molecular and neuronal mechanisms at play, and test new ADHD treatments.

Due to the defects in DAT, DAT-KO mice exhibit spontaneous hyperlocomotion [22, 24]. DAT-KO mice showed an elevation in hyperactivity and velocity, along with less time of immobility, with a breakdown or failing in habituating over time in the open field. DAT-KO mice also buried fewer marbles than respective controls of DAT wild-type (DAT-WT) and -heterozygous (HET) mice in appraisal of obsessive or compulsive-like behaviors, likely because of severe hyperactivity and related attention deficit [25] representing attention deficit hyperactivity (ADHA)-related phenotypes.

Multiple studies have reported a relationship between DAT variants and ADHD [26–28]. It was previously recognized that in patients with ADHD, psychostimulants might interact with the DAT: for example, amphetamine and methylphenidate drugs improve behavioral deficits. However, no clear indication of decreased DAT was identified in patients with ADHD when different researchers compared models to patients, but an increment in the DAT level was seen in the striatum of adults and children [29, 30].

*DAT* is one of the dopamine-related genes that is known to be a candidate for ADHD risk [31] and is also included in the Na+/Cl- dependent transporter family that uptakes dopamine into neurons [20]. DA reuptake by the DAT mainly occurs from the synaptic cleft to the presynaptic terminal and plays an important role in the functioning of the dopamine system [32].

DAT is predominantly expressed in nigrostriatal and mesolimbic dopaminergic neurons of the central nervous system, with the highest levels in the striatum and nucleus accumbens (NAc) [33]. In addition, subcellular ultrastructural studies have confirmed that most DAT in striatal dopamine axons are disseminated at the synaptic periphery and nonsynaptic membrane regions [34].

DAT deficiency in DAT-KO mice results in changes in the DA system. Compared to WT mice, DAT-KO mice had a five-fold higher extracellular DA concentration [23], which is consistent with the 300-fold slower DA clearance in DAT-KO mice [35]. It was also confirmed that electrically stimulated dopamine release in DAT-KO mice was reduced by approximately 75% compared with that in WT controls [36]. Giros et al. [22] reported a quantitative in situ hybridization study that confirmed a reduction in the messenger ribonucleic acid (mRNA) levels of postsynaptic DA receptors D1 and D2, which were further downregulated by almost 50% in the striatum [22]. These studies have shown that the release of dopamine and its receptors is controlled in the brain. Regarding physiological functions, DAT-KO mice had a significantly slower breathing rate with extended inspiration time. DAT-KO mice show a decreased response to hypoxia compared to WT mice; however, CO2 production is unaffected in the mutants [37]. Body temperature of DAT-KO mice doesn’t follow a circadian variation. Circadian analysis revealed a decrease in body temperature during the daytime in DAT-KO mice. The exclusion of DAT in DAT-KO mice resulted in delayed weight gain compared to HET and WT mice. Females without DAT exhibited poor lactation and diminished ability to care for their young. Deletion of DAT causes anterior pituitary hypoplasia and a number of changes in the hypothalamo-pituitary axis characteristics, emphasizing the function of hypothalamic DA reuptake in developmental events [35].

DAT-KO mice exhibit ADHD-related behavioral changes in various psychological experiments [38, 39]. In the open field test, the movement speed and hyperactivity of DAT-KO mice increased, whereas the immobility time decreased [40]. Fewer marbles were buried during the marble-burying test, which was attributed to hyperactivity and inattention [41, 42]. In the cliff avoidance reaction test, it was confirmed that they showed slightly more impulsive behavior, unlike WT mice, which tried to avoid falling [43]. In addition, behavioral experiments such as -the Y-maze and pre-pulse inhibition have confirmed: poor attention, learning, and memory [44–46]. Moreover, very poor learning and memory abilities were confirmed through an eight-arm maze, novel object recognition task, and social food preference transmission tests [35, 47]. Contradictory results were also observed in these animals, such as- the alleviation of hyperactivity by amphetamine, methylphenidate, and cocaine- which act on DAT [23, 48, 49]; further suggesting that the effects of these compounds in ADHD do not target the DA system alone. In addition, methylphenidate-induced increases in DA concentration in the synaptic cleft were not observed in DAT-KO mice [35], suggesting that the reduction in hyperactivity in DAT-KO mice could be due to the targeting of noradrenergic systems other than the dopaminergic system.

As per the results of various studies, DAT impairment could lead to ADHD-related behaviors- such as in multiple genetic studies that revealed the association between DAT gene mutations in ADHD patients [18]; and further brain imaging studies also showed a reduction in DAT levels in ADHD patients [50]. However, other studies have confirmed the opposite results, such as an increase in DAT levels in the striatum of patients [51–53]. Therefore, the specific role of DAT in ADHD pathogenesis remains unclear. However, the DAT-KO mouse model is by far the most well-known and reliable ADHD model, and has provided several clues about the function of this gene, which may be related to psychological disturbances.

### Spontaneously hypertensive rat (SHR) model

Spontaneously hypertensive (SH) rats are one of the most studied animal models of ADHD. This model characterization is based on several findings which recommend that the SHR model could be possibly one of the promising hyperactive model for studying ADHD [54, 55]. The SHR strain was developed by Okamoto and Aoki (1963) [56]. They obtained F1 by crossing male Wistar rats with spontaneous hypertension and females with moderately high blood pressure, and selected and mated the hypertensive rats again, eventually reaching F3, where almost 100% of the rats had spontaneous hypertension [56]. SHRs and Wistar-Kyoto (WKY) controls differed in their home-cage circadian activities, with SHRs being more active than WKYs at numerous time points. Interindividual variance in impulsivity was virtually absent in the WKY strain during the test; however, SHRs showed significant inter individual variability [57].

Generally, in the preliminary stage, the SHR model was developed to study patients with hypertension and related comorbidities in animal settings [56]. Nevertheless, Sagolden et al. reported resultant hyperactivity and spontaneous motor activity during experiments, suggesting that this animal could be used as a model for ADHD [58].

According to several studies, the SHR validated the key symptoms of ADHD, such as attention deficit, hyperactivity, and impulsiveness [59–65]. SHR have been demonstrated to be similar to children with ADHD [66] in that they are more sensitive to delays in reinforcement, which is consistent with a steeper gradient of delays in reinforcement observed in SHR compared to controls [64, 67]. Aase et al. (2006) and Aase and Sagvolden (2005) found higher intraindividual variability in SHR behavior than in controls [68, 69]. This is similar to that observed in children with ADHD [61, 63, 68–70].

Experiential alterations or deficits have been found to be directly associated with frontostriatal system dysfunction. References [54, 71] previously showed that the impaired release of DA was witnessed in SHR in specific areas- clearly observed as affected regions in ADHD, namely: the prefrontal caudate-putamen cortex and NAc [71]. In young male SHRs, D5 and D1 receptor density is typically increased in the neostriatum and NAc -according to a previous study by ref. [72]; which demonstrated that the prefrontal cortex (PFC) of SHR has decreased expression of the D4 receptor gene. Furthermore, alterations in noradrenergic system release have been observed in the PFC and LC (locus coeruleus) [73]. In other words, the noradrenergic system is overactive in the prefrontal cortex of SHR. The production of noradrenaline (NA) in the prefrontal cortex induced by glutamatergic stimulation is elevated in SHRs compared to their respective control WKY rats [74]. Collectively, from the above studies, we recommend SHR as a favorable model for studying ADHD. However, the modifications to this model that affect hypertension may also function as variables. Although this model is valuable, considering the impact of hypertension on it is also important.

The SHR strain was generated as described by Okamoto and Aoki (1963) [56]. They obtained F1 by crossing male Wistar rats with spontaneous hypertension and females with moderately high blood pressure, and selected and bred hypertensive individuals among them again, eventually reaching F3 to such an extent that almost 100% of the individuals showed spontaneous hypertension [56]. As such, SHR was created for the study of hypertension; however, it shows ADHD symptoms such as : impulsivity, learning and memory deficits, hyperactivity, deficient sustained attention, and increased impulsiveness [63, 75]. For example, SHR were confirmed to be hyperactive compared to control WKY rats in an open field test, and this increased activity was observed in both male and female rats [74]. Moreover, similar to children with ADHD, SHRs are less sensitive to delayed reinforcement and more sensitive to immediate behavioral reinforcement than nonhypertensive WKY control rats [63]. The behavioral responsiveness of SHR mice was altered by psychomotor stimulants such as methylphenidate hydrochloride (ritalin) or d-amphetamine, which treat childhood ADHD with major symptoms of attention problems and hyperkinesis. This is consistent with the clinical findings in children with ADHD [76]. In addition, behavioral deficits, such as hyperactivity and impulsiveness, can be alleviated by monoaminergic agents [77, 78].

An in vitro superfusion technique revealed that depolarization (25 mM K1)induced the release of DA from the NAc slices of SHR, which was significantly lower than that in WKY controls [79]. Compared to WKY rats, electrical stimulation required less [3 H]DA in the PFC and caudate-putamen slices of SHR [80]. Miller et al. reported that SHR/NCrl exhibited decreased KCl-evoked DA release versus the WKY/NCrl model of inattentive subtype (ADHD-PI) in the dorsal striatum (Str). The SHR/NCrl model of ADHD-PI showed quicker DA uptake in the ventral Str and NAc compared to both control strains, but the WKY/NCrl model of ADHD-PI had faster DA uptake in the NAc compared to the SD control. These findings show that higher surface expression of DA transporters could elucidate the faster DA absorption in the Str and NAc of these ADHD animal models. [81].

Next, SHR exhibited changes in several brain systems. One of them is the dopamine system. Depolarization (25 mM K1)-induced electrical stimulation and KCl-evoked release of dopamine are significantly lower in the NAc, cortex, caudate-putamen, and striatum than in WKY controls [80–82]. In addition, in SHR, D5 and D1 receptor subtypes showed high levels in the NAc and caudate-putamen [83]; and the expression of the D4 receptor gene in the PFC and the protein synthesis of it, was significantly low [72]; and Moreover, in a study of dopamine-related genes (Drd2, Drd4, and Dat1) in WKY and SHR by Mill- who found several mutations in the DAT1 gene, which could explain some of the behavioral differences between the two animals by DAT1 sequence changes [84].

In addition to changes in the dopamine system, changes in the norepinephrine (NE) system have been observed in SHR. Basal NE concentrations were significantly higher in the frontal cortex, LC, A2 nucleus, and substantia nigra of SHR than in WKY [85]. During early development of SHR, we found higher NE uptake in the frontal cortex, cerebellum, and hypothalamus, and reduced [3H]DHA binding, indicating downregulation of beta-adrenergic receptors in these regions [86]. In the induction of NE release through glutamate, SHR showed more release compared to the WKY controls [87], whereas UK 14,304 (alpha 2 agonists- an adrenergic agonist) and neuropeptides showed less NE release inhibition [88]. The inhibition of alpha2-adrenoceptor-mediated NE release was also reduced, suggesting that auto-receptor function in the PFC was disrupted [82]. PNMT, NAT, and 1A-R mRNA expression levels were higher in SHR, and PNMT mRNA in SHR was three-fold higher than in WKY rats. In contrast, for 2A-R the mRNA expression was three-fold lower in the spinal cord [89]. Enhanced DAT was observed in SHR before the onset of hypertension, whereas enhanced DAT and Dl receptors were observed in posthypertensive SH rats. These findings imply that the dopamine system is involved in the pathophysiology and development of hypertension [90].

In conclusion, SHR can be used as a good ADHD research model based on ADHA-related behavioral characteristics and changes in the brain. However, hypertension-related changes in this model can act as a cause or variable of change. Therefore, the effects of high blood pressure on behavioral changes and brain damage should be considered. We have included Table 1 to briefly describe the different models that can be used to study the different symptoms of ADHD in mutant mice, genes involved, and behavioral changes.

**Table 1:** Examining the reliability of various proposed Attention-Deficit Hyperactivity Disorder (ADHD) transgenic animal models the table was modified and adopted from Pena et al. [154].

Mutant mice	Related genes	Behavioral characteristics	References
Dopamine transporter KO mice	Lack of DAT gene (Slc6a3)	Hyperactivity, Impulsivity, Inattention	[25, 35, 43, 44]
Alpha-synuclein lacking mice	Lack of alpha or/and gamma synuclein	Hyperactivity	[144, 147]
Steroid sulfatase deficit mice	Deletion of STS gene because of fusion of X and Y chromosomes	Hyperactivity, Inattention	[95, 97, 98]
Thyroid hormone receptor receptor–beta(1) KI mice	Knock-in of human thyroid hormone β receptor gene	Hyperactivity, Impulsivity, Inattention	[155]
Coloboma mutant mice	Disruption in approximately 20 genes including SNAP − 25 due to mutation in 2 chromosome	Hyperactivity, Impulsivity, Inattention	[112, 123]
Spontaneously hypertensive rat	Inbred strain derived from the Wistar-Kyoto (WK) rat	Hyperactivity, Impulsivity, Inattention	[56, 63, 74]
Dopamine transporter KD mice	Dopamine transporter expression lowered to 10% of wild-type levels	Hyperactivity	[156]
Dopamine D4 receptor KO mice	Lack of DRD4	-	[157]
6-Hydroxydopamine (6-OHDA) lesioned neonatal rat/mice	6-OHDA selectively damages catecholaminergic D4-KO mouse neurons	Hyperactivity	[158, 159]
Alpha-4 beta-2 nicotinic receptor KO mice	Lack of Alpha-4 beta-2 nicotinic receptor	-	[160]
Neurokinin 1 receptor KO mice	Neurokinin 1 receptor (NK1R) or Tacr1 gene functional ablation	Hyperactivity, Impulsivity, Inattention	[161–163]
P35 KO mice	Knock-out of Cdk5-activating cofactor p35	Hyperactivity	[164, 165]
GC-C KO mice	The guanylyl cyclase-C gene has been deleted	Hyperactivity, Impulsivity, Inattention	[166]
Per1 KO mouse	Per1 gene mouse with targeted mutation (inactivation)	Hyperactivity, Impulsivity	[167]
PI3Kγ KO mouse	Absence of class IB phosphoinositide 3-kinases (PI3Kγ)	Hyperactivity, Inattention	[168]
CK1δ OE mouse	Overexpression of the casein kinase 1 (CK1) subunit in the forebrain	Hyperactivity	[169]
GAT1 KO mouse	Absence of gamma-aminobutyric acid transporter1 (GAT1) gene	Hyperactivity, Impulsivity, Inattention	[170, 171]
nAChR β2 KO mouse	Removal of the gene that codes for the β2-subunit of the nicotinic acetylcholine receptor	Hyperactivity, Impulsivity, Inattention	[160, 172, 173]
ADF/n-cofilin KO mouse	Absence of both actin depolymerizing factor (ADF) and n-cofilin	Hyperactivity, Impulsivity	[174]
GIT1 KO mouse	Loss of the G-protein coupled receptor kinase interacting protein 1 (GIT1) gene	Hyperactivity	[175]
DGKβ KO mouse	Loss of the DGKβ (Dgkb) gene	Hyperactivity, Inattention	[176, 177]
Gβ5 KO mouse	Missing of the type 5 G protein beta subunit (Gβ5) gene	Hyperactivity	[178]
Fmr1-KO mouse	Loss of the fragile X mental retardation 1 (Fmr1) gene	Hyperactivity, Impulsivity, Inattention	[179, 180]
Ptchd1-KO mouse	Inactivation of the Ptchd1 gene	Hyperactivity, Inattention	[181, 182]
NOS1-KO mouse	Neuronal nitric oxide synthase (Nos1) gene ablation	Hyperactivity, Impulsivity	[183]
mAChR M1-KO mouse	Loss of the gene that encrypts for the M1 subtype of the receptor for muscarinic acetylcholine	Hyperactivity	[184, 185]
Brinp1-KO mouse	Absence of the bone morphogenetic protein (BMP) / retinoic acid (RA)-inducible neural-specific protein 1 (BRINP1)	Hyperactivity	[186, 187]
Cdh13-KO mouse	Genetic ablation of the cadherin-13 (Cdh13) gene	Hyperactivity	[188, 189]
DAT-CI	Triple point-mutation in the cocaine-binding site of DAT	Hyperactivity	[190]
BAC DAT-tg	Overexpression of dopamine transporter	-	[191]
Naples high-excitability rat	Lower expression of DA D1 receptor transcripts in NHE, 26 mRNAs greatly expressed in the PFc of NHE rats	Hyperactivity, Inattention	[192, 193]
Acallosal mouse strain	Inbred acallosal mouse strain I/LnJ	Hyperactivity, Impulsivity	[194]
Atxn7 OE mouse	Atxn7 overexpressing	Hyperactivity, Impulsivity	[195]
5HT2C receptor-KO mice	X-chromosome linked serotonin 2c receptor (5HT2C) gene (Htr2c)	Impulsivity	[196]
COMT-KO mice	Catechol-O-methyltransferase (COMT)- KO	Impulsivity	[197]
NF1-KO mice	Neurofibromatosis type 1 (NF1)-KO mice	Inattention	[198]
Nrg3-KO mice	Neuregulin-3 (Nrg3)	Impulsivity	[199]

### Steroid sulfatase

Steroid sulfatase (STS) is an enzyme encoded by the X-linked gene STS in humans and the pseudoautosomal gene STS in mice [91]. STS functions in the desulfation of neurosteroids by hydrolyzing dehydroepiandrosterone sulfate (DHEA-S) to DHEA [92]. DHEA-S and DHEA act as negative regulators of GABA A receptors and positive regulators of NMDA-receptors [93, 94]. STS expression has been confirmed in brain regions important for attention and impulsivity, which are thought to be problematic in ADHD, such as the PFC, thalamus, and basal nucleus [95].

The X and Y chromosomes are joined end-to-end by pseudoautosomal regions in a single large sex chromosome of 39XY*O mice. All other X and Y genes are present in their normal complement despite the deletion of the STS gene [96]. STS is expressed in key areas of the developing brain that are vital for attention and impulsivity as well as in the frontal cortex, thalamus, and basal ganglia. However, the aforementioned regions are likely to be dysregulated in ADHD [95].

Several studies have shown that 39XY*O mice exhibit ADHD-related behaviors, such as hyperactivity, inattention, and occasional aggression [97–99] which have been linked to an increase in serotonin (5-hydroxytryptamine,5-HT) levels in the striatum and hippocampus as a consequence of decreased DHEA [98]. Trent et al. showed that 39XY*O mice had higher ratios for progressive ratio (PR) task thought to index motivation compared to WT mice. However, no variation were observed between the two groups in the behavioral tasks that were thought to index compulsivity [99]. A neurobiological explanation for the behavioral differences between 40,XY and 39,X(Y)*O mice is the regionally specific perturbations of the 5-HT system, which are associated with significant correlations between hippocampal 5-HT levels and PR performance, as well as between striatal 5-HT levels and locomotor activity. These findings imply that functional variations and inactivating mutations within STS may affect ADHD vulnerability and disease endophenotypes by altering the serotonergic system.

Therefore, although 39XY*O mice have some validity as ADHD models based on ADHD-related behavioral phenotypes and altered serotonergic systems, more evidence is needed to establish them as ADHD models.

There is a male bias in ADHD [100]. According to previous reports by Szatmari et al. and Gomez et al., the male-to-female ratios were 3:1 and 5:1, respectively [101, 102]. In addition, if there is a male bias, the association between the X-linked gene and ADHD may also be reflected, as indicated by several studies reporting that patients with Xp deletions exhibit ADHD-like cognitive-behavioral characteristics [103, 104].

Moreover, female with Turners syndrome (45XO) who are haplo-insufficient for genes which results in the escape of X-inactivation- further establish that with different cognitive deficits consisting of: social, visuospatial, memory, cognitive and attentional deficits [105, 106].

Kent et al. (2008) reported the neurobehavioral characteristics of 25 boys with X-linked Ichthyosis, a genetic skin disorder caused by deletion or point mutation of the STS gene, confirming the diagnostic and statistical manual of mental disorders IV ADHD with no comorbidity: 32% (8 cases) of patients were diagnosed with the inattentive subtype [107]. ADHD has been found in boys with both STS deletions and putative point mutations, indicating that STS insufficiency may be the cause of the high risk of inattentive symptoms in these populations [100].

### Coloboma mice

Coloboma mutant mice were first described by Searle et al., developed through irradiation caused by a mutation on chromosome 2 [108] and reported to be mutated by approximately 20 genes [109–111] such as: phospholipase C beta-1 (Plcb1), jagged 1 (Jag1), and synaptosomal-associated protein 25 kDa (Snap25). Among all the genetic disarrangements, SNAP25 gene is attracting attention owing to its association with ADHD in terms of pathophysiology [112–116].

SNAP25 is a component of the SNARE (soluble N-ethylmaleimide-sensitive factor attachment protein receptor) complex, which facilitates the fusion and docking of postsynaptic vesicles to enable the release of neurotransmitters [117, 118]. It was previously shown that variations in the SNAP-25 gene could lead to symptoms of ADHD by altering the levels of dopamine and other neurotransmitters at the synapse [119].

SNAP25 dysfunction causes changes in the dopaminergic system. Coloboma mutant mice exhibit a marked decrease in dopamine release from the dorsal striatum compared to their respective controls [120]. In addition, mRNA expression of the dopamine D2 receptor increased in the ventral tegmental area and substantia nigra, which is consistent with the inhibition of dopamine neurons [121, 122]. In addition to the dopamine system, SNAP25 anomalies result in altered NE elevations in the noradrenergic system [121]. The reduction of NE in mice with N-(2-chloroethyl)-N-ethyl-2-bromobenzylamine hydrochloride reduced hyperactivity but did not improve impulsivity, demonstrating a link between the noradrenergic system and hyperactivity in this model [123, 124]. In a coloboma mouse study, Bruno et al (2006) discovered that the alpha (2 C)-adrenergic receptor (ADRA2C) was involved in hyperactivity [125].

Hence, these synaptic differences in coloboma mutant mice can serve as the foundation for the basic approval of this model for triggering behavioral anomalies such as hyperactivity, inattention, and impulsivity. This mice model displayed spontaneous locomotor hyperactivity in an open-field experiment [126] and less patience than the control group in a delayed reinforcement task, demonstrating the characteristics of inattention and impulsivity [123].

Studies have suggested that coloboma mutant mice show a reduction in hyperactivity with d-amphetamine and not with methylphenidate; therefore, it works as a moderate to conventional ADHD treatment [109, 110, 127]. Taking all these considerations into account, we propose that coloboma mutant mice or Snap25-mutant mice could be used as promising models for ADHD.

### Alpha-synuclein lacking mice

Alpha-, beta-, and gamma-synucleins belong to the synuclein family and are small, soluble proteins that have been found only in vertebrates and are expressed in nerve tissues and some tumors [128]. Among these, mutations in alpha-synuclein are associated with rare familial cases of Parkinson’s disease as well as the accumulation of this protein in AD and several neurodegenerative diseases [129, 130]. Alpha-synuclein is predominantly expressed in the brain tissues of the neocortex, hippocampus, striatum, thalamus, and cerebellum, and is found at presynaptic terminals [131]. The human and rodent sequences were 95.3% identical except of six amino acids [132]. The amino acid residue at position 53 is typically alanine in humans and threonine in rodents. Surprisingly, the same change, Ala-53-Thr, has been found in some family cases of Parkinson’s disease (PD) [129].

Phospholipase D2 (PLD2) has been reported to function in cytoskeletal regulation and/or endocytosis [133]. It has been reported that a- and b synuclein can selectively inhibit PLD2 through direct interaction on the membrane surface, suggesting that synuclein may play an important role in regulating the vesicle transport process [134]. PLD2 overexpression in the rat substantia nigra pars compacta (SNc) results in: severe neurodegeneration of DA neurons, loss of striatal DA, and ipsilateral amphetamine-induced rotational asymmetry [135]. Other studies have found that alpha-synuclein expression in the rat brain-especially in the cerebral cortex, hippocampus, and dentate gyrus- is related to the localization of molecules associated with the phosphoinositol (PI) secondary messenger pathway, such as phospholipase C1 (PLC1) and muscarinic cholinergic receptor types m1 and m3 [136]. This discovery gave them the idea that alpha-synuclein could be involved in synaptic vesicle release and/or recycling in response to PI stimulation. This notion is supported by the discovery that a-synuclein can bind to tiny, unilamellar phospholipid vesicles. [137]. Therefore, alpha-synuclein may also play a role in the dopamine system because it is associated with Parkinson’s disease, along with functions related to the vesicles of alpha-synuclein.

In fact, the depletion of alpha-synuclein in primary hippocampal neurons treated with antisense oligonucleotides reduces the pool of presynaptic vesicles [138]. Several other studies have suggested that alpha-synuclein is involved in the regulation of DA homeostasis [139, 140]. Tissue cultures have shown that alpha-synuclein inhibits DA synthesis by regulating the activity of tyrosine hydroxylase, protein phosphatase 2 A, and aromatic amino acid decarboxylase [141–143]. Alpha-synuclein KO mice, in contrast, showed increased dopamine release in the nigrostriatal terminals as a result of paired electrical stimuli- indicating that alpha-synuclein acts as a negative regulator of DA neurotransmission [144]. Alpha-synuclein KO mice showed a reduced effect of D-amphetamine compared to WT, further supporting the fact that alpha-synuclein is a negative regulator of DA neurotransmission [144].

Despite these changes in the dopamine system, behavioral phenotypes related to ADHD are not easily observed in alpha-synuclein-related mutant mice. When comparing alpha-synuclein-lacking mice and WT mice, no significant difference in amphetamine-induced activity was observed, and the rearing was the same [145]. In a study on the association between alpha-synuclein and anxiety, no significant difference was observed between alpha-synuclein knockout and WT mice in emotionality tests, such as the open field, elevated plus maze, and light–dark box. Therefore, alpha-synuclein is not involved in anxiety in mice [146]. In Senior’s study, significant results were found, and it was confirmed that alpha-synuclein and gamma-synuclein double-null mice were hyperactive in the novel environment and alternated at a lower rate in the T-maze spontaneous alternation task. In addition, the concentration of extracellular DA in the striatum doubled in double-null mice after discrete electrical stimulation [147]. However, this does not only target alpha-synuclein. Although the behavioral characteristics of hyperactivity have been identified, studies on other ADHD-related behavioral phenotypes-such as inattention and anxiety- are lacking. Therefore, further studies are needed using alpha-synuclein-deficient mice as ADHA-related models. As related behavioral phenotypes and changes in the dopamine system were confirmed in double-null mice; if the study was expanded to the synuclein family rather than limited to alpha-synuclein, it may provide another clue to research its relationship with ADHD.

## Conclusion and future prospective

Animal models are vital research tools that may help us better understand the possible complex mechanisms involved in the development of a disease and enable us to screen and report new effective medications for therapy that can be translated to humans. Animal models of ADHD are categorized as perfect mimics of all disease-inducing features at both the behavioral and physiological levels. Abundant evidence regarding the genetics of ADHD exists; however, these findings appear to be inconsistent. Studies have shown that ADHD may develop from the interaction of many polygenic genes. Mooney et al. combined the results of numerous analysis methods to determine the pathways most likely to be connected with ADHD as well as to evaluate different types of route methodologies and the benefits involved in this approach [148].

This study acknowledges seven pathways, including :RhoA signaling, glycosaminoglycan biosynthesis, fibroblast growth factor receptor activity, and pathways containing potassium channel genes, reported as nominally significant by multiple analysis methods using two GWAS databases. This study confirmed earlier beliefs regarding how controlling neurotransmitters, neurite outgrowth, and axon regulation contribute to the ADHD phenotype; and stressed the importance of cross-method convergence when evaluating route analysis results. The polygenic model of illness risk was consistent with the excess minor SNP effects found in each of these pathways. These pathway correlations offer additional support for earlier hypotheses concerning the etiology of ADHD, particularly those associated with the regulation of neurotransmitter release and neurodevelopmental processes; however, further studies are required to confirm this hypothesis.

To study the mechanism and to understand the etiopathology of ADHD studies have highlighted the importance of using induced pluripotent stem cells (iPSCs) for disease modeling. This method allows us to analyze individual-specific neuronal cell lines in vitro in order to research cellular malfunction and identify the underlying genetic variables [149–151]. Reference [152] developed a methodology for generating iPSCs from hair-derived keratinocytes as beginning somatic cells from patients in order to circumvent the invasive aspect of sample collection in the research of early neurodevelopment diseases such as ADHD [152].

Another pathway discussed by Ohki et al. regarding the cause and pathophysiology of ADHD supported the hypothesis of the Wnt and mTOR signaling pathways [153]. Cellular proliferation, polarity, and differentiation are controlled by the Wnt signaling system, whereas synaptic plasticity and several other important neurodevelopmental processes are controlled by the mTOR pathway. Therefore, dysregulation of these time-dependent pathways may result in neurodevelopmental delays and ADHD phenotypes.

Additional genetic variations are present in other models (dopamine transporter gene knockout mice, coloboma mice, Naples hyperexcitable rats, steroid sulfatase, alpha-synuclein-lacking mice, and neonatal lesioning of dopaminergic neurons with 6-hydroxydopamine). However, none of them are fully comparable to clinical ADHD. The pathophysiology involved varies, including both deficient and excessive dopaminergic functioning, and there is probable involvement of other monoamine neurotransmitters such as dopamine, serotonin, and noradrenaline. Therefore, improved models as well as further testing of their ability to predict treatment responses are required. Some aspects of ADHD behavior may result from an imbalance between increased noradrenergic activity and decreased dopaminergic regulation of neural circuits that involve the prefrontal cortex. In addition to providing unique insights into the neurobiology of ADHD, animal models are also used to test new drugs that can alleviate ADHD symptoms.

The evidence addressed in this study suggests that currently available animal models may be useful for studying human behavioral disorders. Furthermore, our current knowledge of ADHD neurobiology is insufficient, making it challenging to identify an optimal model for investigating ADHD.

## References

[1] Prakash J, Chatterjee K, Guha S, Srivastava K, Chauhan VS (2021). Adult attention-deficit Hyperactivity disorder: from clinical reality toward conceptual clarity. Ind Psychiatry J.

[2] Abdelnour E, Jansen MO, Gold JA (2022). ADHD diagnostic trends: increased recognition or overdiagnosis?. Mo Med.

[3] Renner TJ, Gerlach M, Romanos M, Herrmann M, Reif A, Fallgatter AJ (2008). Neurobiology of attention-deficit hyperactivity disorder. Nervenarzt.

[4] Lycett K, Mensah FK, Hiscock H, Sciberras E (2014). A prospective study of sleep problems in children with ADHD. Sleep Med.

[5] Tsai FJ, Tseng WL, Yang LK, Gau SS (2019). Psychiatric comorbid patterns in adults with attention-deficit hyperactivity disorder: treatment effect and subtypes. PLoS One.

[6] Villemonteix T, De Brito SA, Slama H, Kavec M, Balériaux D, Metens T (2015). Grey matter volume differences associated with gender in children with attention-deficit/hyperactivity disorder: a voxel-based morphometry study. Dev Cogn Neurosci.

[7] Forssman L, Eninger L, Tillman CM, Rodriguez A, Bohlin G (2012). Cognitive functioning and family risk factors in relation to symptom behaviors of ADHD and ODD in adolescents. J Atten Disord.

[8] Devlin B, Kelsoe JR, Sklar P, Daly MJ, O’Donovan MC, Craddock N (2013). Genetic relationship between five psychiatric disorders estimated from genome-wide SNPs. Nat Genet.

[9] Banerjee TD, Middleton F, Faraone SV (2007). Environmental risk factors for attention‐deficit hyperactivity disorder. Acta Paediatr.

[10] De Zeeuw P, Zwart F, Schrama R, Van Engeland H, Durston S (2012). Prenatal exposure to cigarette smoke or alcohol and cerebellum volume in attention-deficit/hyperactivity disorder and typical development. Transl Psychiatry.

[11] Millichap JG, Yee MM (2012). The diet factor in attention-deficit/hyperactivity disorder. Pediatrics.

[12] Galvez-Contreras AY, Campos-Ordonez T, Gonzalez-Castaneda RE, Gonzalez-Perez O (2017). Alterations of growth factors in autism and attention-deficit/hyperactivity disorder. Front psychiatry.

[13] da Silva BS, Grevet EH, Silva LCF, Ramos JKN, Rovaris DL, Bau CHD (2023). An overview on neurobiology and therapeutics of attention-deficit/hyperactivity disorder. Discov Ment Health.

[14] Slobodin O, Davidovitch M (2019). Gender differences in objective and subjective measures of ADHD among clinic-referred children. Front Hum Neurosci.

[15] Seo J-C, Jon D-I, Shim S-H, Sung H-M, Woo YS, Hong J (2022). Prevalence and comorbidities of attention deficit hyperactivity disorder among adults and children/adolescents in Korea. Clin Psychopharmacol Neurosci.

[16] Kohe SE, Gowing EK, Seo S, Oorschot DE (2023). A Novel Rat Model of ADHD-like hyperactivity/impulsivity after delayed reward has selective loss of dopaminergic neurons in the right ventral tegmental area. Int J Mol Sci.

[17] McKinney Jr WT. Models of mental disorders: a new comparative psychiatry. Springer Science & Business Media; 2012.

[18] McKinney W. Basis of development of animal models in psychiatry: an overview. In: Koob GF, Ehlers CL, Kupfer DJ, editors. Animal models of depression; 1989. p. 3–17, Birkhauser, Boston, Basel.

[19] Willner P (1984). The validity of animal models of depression. Psychopharmacology.

[20] Reith ME, Xu C, Chen N-H (1997). Pharmacology and regulation of the neuronal dopamine transporter. Eur J Pharmacol.

[21] Amara SG, Kuhar MJ (1993). Neurotransmitter transporters: recent progress. Annu Rev Neurosci.

[22] Giros B, Jaber M, Jones SR, Wightman RM, Caron MG (1996). Hyperlocomotion and indifference to cocaine and amphetamine in mice lacking the dopamine transporter. Nature.

[23] Jones SR, Gainetdinov RR, Jaber M, Giros B, Wightman RM, Caron MG (1998). Profound neuronal plasticity in response to inactivation of the dopamine transporter. Proc Natl Acad Sci.

[24] Sora I, Wichems C, Takahashi N, Li X-F, Zeng Z, Revay R (1998). Cocaine reward models: conditioned place preference can be established in dopamine-and in serotonin-transporter knockout mice. Proc Natl Acad Sci.

[25] Fox MA, Panessiti MG, Hall FS, Uhl GR, Murphy DL (2013). An evaluation of the serotonin system and perseverative, compulsive, stereotypical, and hyperactive behaviors in dopamine transporter (DAT) knockout mice. Psychopharmacol.

[26] Faraone S, Spencer T, Madras B, Zhang-James Y, Biederman J (2014). Functional effects of dopamine transporter gene genotypes on in vivo dopamine transporter functioning: a meta-analysis. Mol Psychiatry.

[27] Faraone SV, Bonvicini C, Scassellati C (2014). Biomarkers in the diagnosis of ADHD–promising directions. Curr Psychiatry Rep..

[28] Bonvicini C, Faraone S, Scassellati C (2016). Attention-deficit hyperactivity disorder in adults: a systematic review and meta-analysis of genetic, pharmacogenetic and biochemical studies. Mol Psychiatry.

[29] Fernández-Jaén A, López-Martín S, Albert J, Fernández-Mayoralas DM, Fernández-Perrone AL, de La Peña MJ (2015). Cortical thickness differences in the prefrontal cortex in children and adolescents with ADHD in relation to dopamine transporter (DAT1) genotype. Psychiatry Res.

[30] Hoogman M, Onnink M, Cools R, Aarts E, Kan C, Vasquez AA (2013). The dopamine transporter haplotype and reward-related striatal responses in adult ADHD. Eur Neuropsychopharmacol.

[31] Cook EH, Stein MA, Krasowski MD, Cox NJ, Olkon DM, Kieffer JE (1995). Association of attention-deficit disorder and the dopamine transporter gene. Am J Hum Genet.

[32] Vaughan RA, Foster JD (2013). Mechanisms of dopamine transporter regulation in normal and disease states. Trends Pharm Sci.

[33] Ciliax BJ, Heilman C, Demchyshyn LL, Pristupa ZB, Ince E, Hersch SM (1995). The dopamine transporter: immunochemical characterization and localization in brain. J Neurosci.

[34] Nirenberg MJ, Chan J, Pohorille A, Vaughan RA, Uhl GR, Kuhar MJ (1997). The dopamine transporter: comparative ultrastructure of dopaminergic axons in limbic and motor compartments of the nucleus accumbens. J Neurosci.

[35] Gainetdinov RR, Jones SR, Caron MG (1999). Functional hyperdopaminergia in dopamine transporter knock-out mice. Biol Psychiatry.

[36] Jones SR, Gainetdinov RR, Wightman RM, Caron MG (1998). Mechanisms of amphetamine action revealed in mice lacking the dopamine transporter. J Neurosci.

[37] Efimova EV, Gainetdinov RR, Budygin EA, Sotnikova TD (2016). Dopamine transporter mutant animals: a translational perspective. J Neurogenet.

[38] Seibenhener ML, Wooten MC. Use of the open field maze to measure locomotor and anxiety-like behavior in mice. J Vis Exp. 2015. 10.3791/52434.e52434. 10.3791/52434 PMC4354627 25742564

[39] Sontag TA, Tucha O, Walitza S, Lange KW (2010). Animal models of attention deficit/hyperactivity disorder (ADHD): a critical review. ADHD Atten Deficit Hyperactivity Disord.

[40] Spielewoy C, Roubert C, Hamon M, Nosten M, Betancur C, Giros B (2000). Behavioural disturbances associated with hyperdopaminergia in dopamine-transporter knockout mice. Behav Pharmacol.

[41] Angoa-Pérez M, Kane MJ, Briggs DI, Francescutti DM, Kuhn DM. Marble burying and nestlet shredding as tests of repetitive, compulsive-like behaviors in mice. J Vis Exp. 2013. 10.3791/50978.50978. 10.3791/50978 PMC4108161 24429507

[42] Torres-Lista V, López-Pousa S, Giménez-Llort L (2015). Marble-burying is enhanced in 3xTg-AD mice, can be reversed by risperidone and it is modulable by handling. Behav Process.

[43] Yamashita M, Sakakibara Y, Hall FS, Numachi Y, Yoshida S, Kobayashi H (2013). Impaired cliff avoidance reaction in dopamine transporter knockout mice. Psychopharmacology.

[44] Li B, Arime Y, Hall FS, Uhl GR, Sora I (2010). Impaired spatial working memory and decreased frontal cortex BDNF protein level in dopamine transporter knockout mice. Eur J Pharm.

[45] Ralph RJ, Paulus MP, Fumagalli F, Caron MG, Geyer MA (2001). Prepulse inhibition deficits and perseverative motor patterns in dopamine transporter knock-out mice: differential effects of D1 and D2 receptor antagonists. J Neurosci.

[46] Yamashita M, Fukushima S, Shen H-w, Hall FS, Uhl GR, Numachi Y (2006). Norepinephrine transporter blockade can normalize the prepulse inhibition deficits found in dopamine transporter knockout mice. Neuropsychopharmacology.

[47] Wong P, Chang CCR, Marx CE, Caron MG, Wetsel WC, Zhang X (2012). Pregnenolone rescues schizophrenia-like behavior in dopamine transporter knockout mice. PloS one.

[48] Gainetdinov RR, Jones SR, Fumagalli F, Wightman RM, Caron MG (1998). Re-evaluation of the role of the dopamine transporter in dopamine system homeostasis. Brain Res Rev.

[49] Gainetdinov RR, Mohn AR, Bohn LM, Caron MG (2001). Glutamatergic modulation of hyperactivity in mice lacking the dopamine transporter. Proc Natl Acad Sci.

[50] Hesse S, Ballaschke O, Barthel H, Sabri O (2009). Dopamine transporter imaging in adult patients with attention-deficit/hyperactivity disorder. Psychiatry Res: Neuroimaging.

[51] Krause KH, Dresel SH, Krause J, Kung HF, Tatsch K (2000). Increased striatal dopamine transporter in adult patients with attention deficit hyperactivity disorder: effects of methylphenidate as measured by single photon emission computed tomography. Neurosci Lett.

[52] Dougherty DD, Bonab AA, Spencer TJ, Rauch SL, Madras BK, Fischman AJ (1999). Dopamine transporter density in patients with attention deficit hyperactivity disorder. Lancet.

[53] Cheon KA, Ryu YH, Kim YK, Namkoong K, Kim CH, Lee JD (2003). Dopamine transporter density in the basal ganglia assessed with [123I]IPT SPET in children with attention deficit hyperactivity disorder. Eur J Nucl Med Mol Imaging.

[54] Leffa DT, Torres IL, Rohde LA (2019). A review on the role of inflammation in attention-deficit/hyperactivity disorder. Neuroimmunomodulation.

[55] Wang L, Mu Z, Lin X, Geng J, Xiao TQ, Zhang Z (2017). Simultaneous imaging of cerebrovascular structure and function in hypertensive rats using synchrotron radiation angiography. Front Aging Neurosci.

[56] Okamoto K, Aoki K (1963). Development of a strain of spontaneously hypertensive rats. Jpn Circ J.

[57] Adriani W, Caprioli A, Granstrem O, Carli M, Laviola G (2003). The spontaneously hypertensive-rat as an animal model of ADHD: evidence for impulsive and non-impulsive subpopulations. Neurosci Biobehav Rev.

[58] Sagvolden T, Johansen EB, Wøien G, Walaas SI, Storm-Mathisen J, Bergersen LH (2009). The spontaneously hypertensive rat model of ADHD-the importance of selecting the appropriate reference strain. Neuropharmacology.

[59] Johansen EB, Aase H, Meyer A, Sagvolden T (2002). Attention-deficit/hyperactivity disorder (ADHD) behaviour explained by dysfunctioning reinforcement and extinction processes. Behav Brain Res.

[60] Johansen EB, Sagvolden T (2004). Response disinhibition may be explained as an extinction deficit in an animal model of attention-deficit/hyperactivity disorder (ADHD). Behav Brain Res.

[61] Sagvolden T, Russell VA, Aase H, Johansen EB, Farshbaf M (2005). Rodent models of attention-deficit/hyperactivity disorder. Biol Psychiatry.

[62] Sagvolden T, Aase H, Zeiner P, Berger D (1998). Altered reinforcement mechanisms in attention-deficit/hyperactivity disorder. Behav Brain Res.

[63] Sagvolden T (2000). Behavioral validation of the spontaneously hypertensive rat (SHR) as an animal model of attention-deficit/hyperactivity disorder (AD/HD). Neurosci Biobehav Rev.

[64] Johansen EB, Sagvolden T, Kvande G (2005). Effects of delayed reinforcers on the behavior of an animal model of attention-deficit/hyperactivity disorder (ADHD). Behav Brain Res.

[65] van den Bergh FS, Bloemarts E, Chan JS, Groenink L, Olivier B, Oosting RS (2006). Spontaneously hypertensive rats do not predict symptoms of attention-deficit hyperactivity disorder. Pharmacol Biochem Behav.

[66] Sonuga-Barke E, Taylor E, Sembi S, Smith J (1992). Hyperactivity and delay aversion: I. The effect of delay on choice. Child Psychol Psychiatry Allied Discip.

[67] Johansen EB, Killeen PR, Sagvolden T (2007). Behavioral variability, elimination of responses, and delay-of-reinforcement gradients in SHR and WKY rats. Behav Brain Funct.

[68] Aase H, Meyer A, Sagvolden T (2006). Moment-to-moment dynamics of ADHD behaviour in South African children. Behav Brain Funct.

[69] Aase H, Sagvolden T (2005). Moment-to-moment dynamics of ADHD behaviour. Behav Brain Funct.

[70] Russell VA, Oades RD, Tannock R, Killeen PR, Auerbach JG, Johansen EB (2006). Response variability in attention-deficit/hyperactivity disorder: a neuronal and glial energetics hypothesis. Behav Brain Funct.

[71] Leffa, Panzenhagen DT, Salvi AC, Bau CHD AA, Pires, Torres ILS GN (2019). Systematic review and meta-analysis of the behavioral effects of methylphenidate in the spontaneously hypertensive rat model of attention-deficit/hyperactivity disorder. Neurosci Biobehav Rev.

[72] Li Q, Lu G, Antonio G, Mak Y, Rudd JA, Fan M (2007). The usefulness of the spontaneously hypertensive rat to model attention-deficit/hyperactivity disorder (ADHD) may be explained by the differential expression of dopamine-related genes in the brain. Neurochem Int.

[73] Rahi V, Kumar P (2021). Animal models of attention‐deficit hyperactivity disorder (ADHD). Int J Dev Neurosci.

[74] Bayless DW, Perez MC, Daniel JM (2015). Comparison of the validity of the use of the spontaneously hypertensive rat as a model of attention deficit hyperactivity disorder in males and females. Behav Brain Res.

[75] Wyss JM, Fisk G, van Groen T (1992). Impaired learning and memory in mature spontaneously hypertensive rats. Brain Res.

[76] Sagvolden T, Metzger MA, Schiorbeck HK, Rugland A-L, Spinnangr I, Sagvolden G (1992). The spontaneously hypertensive rat (SHR) as an animal model of childhood hyperactivity (ADHD): changed reactivity to reinforcers and to psychomotor stimulants. Behav Neural Biol.

[77] Boix F, Qiao S-w, Kolpus T, Sagvolden T (1998). Chronic L-deprenyl treatment alters brain monoamine levels and reduces impulsiveness in an animal model of Attention-Deficit/Hyperactivity Disorder. Behav Brain Res.

[78] Myers MM, Musty RE, Hendley ED (1982). Attenuation of hyperactivity in the spontaneously hypertensive rat by amphetamine. Behav Neural Biol.

[79] Russell VA (2000). The nucleus accumbens motor-limbic interface of the spontaneously hypertensive rat as studied in vitro by the superfusion slice technique. Neurosci Biobehav Rev.

[80] Russell V, de Villiers A, Sagvolden T, Lamm M, Taljaard J (1998). Differences between electrically-, ritalin-and D-amphetamine-stimulated release of [3H] dopamine from brain slices suggest impaired vesicular storage of dopamine in an animal model of attention-deficit hyperactivity disorder. Behav Brain Res.

[81] Miller EM, Pomerleau F, Huettl P, Russell VA, Gerhardt GA, Glaser PE (2012). The spontaneously hypertensive and Wistar Kyoto rat models of ADHD exhibit sub-regional differences in dopamine release and uptake in the striatum and nucleus accumbens. Neuropharmacology.

[82] Russell V, Allie S, Wiggins T (2000). Increased noradrenergic activity in prefrontal cortex slices of an animal model for attention-deficit hyperactivity disorder — the spontaneously hypertensive rat. Behav Brain Res.

[83] Carey MP, Diewald LM, Esposito FJ, Pellicano MP, Carnevale UAG, Sergeant JA (1998). Differential distribution, affinity and plasticity of dopamine D-1 and D-2 receptors in the target sites of the mesolimbic system in an animal model of ADHD. Behav Brain Res.

[84] Mill J, Xu X, Ronald A, Curran S, Price T, Knight J (2005). Quantitative trait locus analysis of candidate gene alleles associated with attention deficit hyperactivity disorder (ADHD) in five genes: DRD4, DAT1, DRD5, SNAP‐25, and 5HT1B. Am J Med Genet Part B: Neuropsychiatr Genet.

[85] de Villiers AS, Russell VA, Sagvolden T, Searson A, Jaffer A, Taljaard JJ (1995). Alpha 2-adrenoceptor mediated inhibition of [3H]dopamine release from nucleus accumbens slices and monoamine levels in a rat model for attention-deficit hyperactivity disorder. Neurochem Res.

[86] Myers MM, Whittemore SR, Hendley ED (1981). Changes in catecholamine neuronal uptake and receptor binding in the brains of spontaneously hypertensive rats (SHR). Brain Res.

[87] Russell VA, Wiggins TM (2000). Increased glutamate-stimulated norepinephrine release from prefrontal cortex slices of spontaneously hypertensive rats. Metab Brain Dis.

[88] Tsuda K, Tsuda S, Masuyama Y, Goldstein M (1990). Norepinephrine release and neuropeptide Y in medulla oblongata of spontaneously hypertensive rats. Hypertension.

[89] Reja V, Goodchild AK, Pilowsky PM (2002). Catecholamine-related gene expression correlates with blood pressures in SHR. Hypertension.

[90] Watanabe Y, Fujita M, Ito Y, Okada T, Kusuoka H, Nishimura T (1997). Brain dopamine transporter in spontaneously hypertensive rats. J Nucl Med.

[91] Yen PH, Marsh B, Allen E, Tsai SP, Ellison J, Connolly L (1988). The human X-linked steroid sulfatase gene and a Y-encoded pseudogene: evidence for an inversion of the Y chromosome during primate evolution. Cell.

[92] Rižner TL (2016). The important roles of steroid sulfatase and sulfotransferases in gynecological diseases. Front Pharmacol.

[93] Majewska MD, Dermirgören S, London ED (1990). Binding of pregnenolone sulfate to rat brain membranes suggests multiple sites of steroid action at the GABAA receptor. Eur J Pharmacol: Mol Pharmacol.

[94] Reddy DS (2010). Neurosteroids: endogenous role in the human brain and therapeutic potentials. Prog Brain Res.

[95] Stergiakouli E, Langley K, Williams H, Walters J, Williams NM, Suren S (2011). Steroid sulfatase is a potential modifier of cognition in attention deficit hyperactivity disorder. Genes Brain Behav.

[96] Burgoyne P, Mahadevaiah S, Perry J, Palmer S, Ashworth A (1998). The Y* rearrangement in mice: new insights into a perplexing PAR. Cytogenet Cell Genet.

[97] Davies W, Humby T, Kong W, Otter T, Burgoyne PS, Wilkinson LS (2009). Converging pharmacological and genetic evidence indicates a role for steroid sulfatase in attention. Biol Psychiatry.

[98] Trent S, Dennehy A, Richardson H, Ojarikre OA, Burgoyne PS, Humby T (2012). Steroid sulfatase-deficient mice exhibit endophenotypes relevant to attention deficit hyperactivity disorder. Psychoneuroendocrinology.

[99] Trent S, Cassano T, Bedse G, Ojarikre OA, Humby T, Davies W (2012). Altered serotonergic function may partially account for behavioral endophenotypes in steroid sulfatase-deficient mice. Neuropsychopharmacology.

[100] Brookes KJ, Hawi Z, Kirley A, Barry E, Gill M, Kent L (2008). Association of the steroid sulfatase (STS) gene with attention deficit hyperactivity disorder. Am J Med Genet Part B: Neuropsychiatr Genet.

[101] Szatmari P, Offord DR, Boyle MH (1989). Ontario child health study: prevalence of attention deficit disorder with hyperactivity. J Child Psychol Psychiatry.

[102] Gomez R, Harvey J, Quick C, Scharer I, Harris G (1999). DSM-IV AD/HD: confirmatory factor models, prevalence, and gender and age differences based on parent and teacher ratings of Australian primary school children. J Child Psychol Psychiatry.

[103] Tobias ES, Bryce G, Farmer G, Barton J, Colgan J, Morrison N (2001). Absence of learning difficulties in a hyperactive boy with a terminal Xp deletion encompassing the MRX49 locus. J Med Genet.

[104] Doherty MJ, Glass IA, Bennett CL, Cotter PD, Watson NF, Mitchell AL (2003). An Xp; Yq translocation causing a novel contiguous gene syndrome in brothers with generalized epilepsy, ichthyosis, and attention deficits. Epilepsia.

[105] Nijhuis-van der Sanden MW, Eling PA, Otten BJ (2003). A review of neuropsychological and motor studies in Turner Syndrome. Neurosci Biobehav Rev.

[106] Skuse DH (2005). X-linked genes and mental functioning. Hum Mol Genet.

[107] Kent L, Emerton J, Bhadravathi V, Weisblatt E, Pasco G, Willatt LR (2008). X-linked ichthyosis (steroid sulfatase deficiency) is associated with increased risk of attention deficit hyperactivity disorder, autism and social communication deficits. J Med Genet.

[108] Searle AG (1966). Curtailed, a new dominant T-allele in the house mouse. Genet Res.

[109] Hess EJ, Collins KA, Wilson MC (1996). Mouse model of hyperkinesis implicates SNAP-25 in behavioral regulation. J Neurosci.

[110] Wilson MC (2000). Coloboma mouse mutant as an animal model of hyperkinesis and attention deficit hyperactivity disorder. Neurosci Biobehav Rev.

[111] Hess EJ, Collins KA, Copeland NG, Jenkins NA, Wilson MC (1994). Deletion Map of the Coloboma (Cm) Locus on Mouse Chromosome 2. Genomics.

[112] Mill J, Curran S, Kent L, Gould A, Huckett L, Richards S (2002). Association study of a SNAP‐25 microsatellite and attention deficit hyperactivity disorder. Am J Med Genet.

[113] Barr C, Feng Y, Wigg K, Bloom S, Roberts W, Malone M (2000). Identification of DNA variants in the SNAP-25 gene and linkage study of these polymorphisms and attention-deficit hyperactivity disorder. Mol psychiatry.

[114] Condliffe SB, Corradini I, Pozzi D, Verderio C, Matteoli M (2010). Endogenous SNAP-25 regulates native voltage-gated calcium channels in glutamatergic neurons. J Biol Chem.

[115] Liu Y, Ma P, Cassidy PA, Carmer R, Zhang G, Venkatraman P (2017). Statistical analysis of zebrafish locomotor behaviour by generalized linear mixed models. Sci Rep..

[116] Wang L, He F, Bu J, Liu X, Du W, Dong J (2012). ABCB6 mutations cause ocular coloboma. Am J Hum Genet.

[117] Bajjalieh SM, Frantz G, Weimann JM, McConnell SK, Scheller R (1994). Differential expression of synaptic vesicle protein 2 (SV2) isoforms. J Neurosci.

[118] McMahon HT, Missler M, Li C, Südhof TC (1995). Complexins: cytosolic proteins that regulate SNAP receptor function. Cell.

[119] Mill J, Richards S, Knight J, Curran S, Taylor E, Asherson P (2004). Haplotype analysis of SNAP-25 suggests a role in the aetiology of ADHD. Mol Psychiatry.

[120] Raber J, Mehta PP, Kreifeldt M, Parsons LH, Weiss F, Bloom FE (1997). Coloboma hyperactive mutant mice exhibit regional and transmitter-specific deficits in neurotransmission. J Neurochem.

[121] Jones M, Williams M, Hess E (2001). Abnormal presynaptic catecholamine regulation in a hyperactive SNAP-25-deficient mouse mutant. Pharmacol Biochem Behav.

[122] Jones MD, Williams ME, Hess EJ (2001). Expression of catecholaminergic mRNAs in the hyperactive mouse mutant coloboma. Mol Brain Res.

[123] Bruno KJ, Freet CS, Twining RC, Egami K, Grigson PS, Hess EJ (2007). Abnormal latent inhibition and impulsivity in coloboma mice, a model of ADHD. Neurobiol Dis.

[124] Jones MD, Hess EJ (2003). Norepinephrine regulates locomotor hyperactivity in the mouse mutant coloboma. Pharmacol Biochem Behav.

[125] Bruno KJ, Hess EJ (2006). The α2C-adrenergic receptor mediates hyperactivity of coloboma mice, a model of attention deficit hyperactivity disorder. Neurobiol Dis.

[126] Hess E, Jinnah H, Kozak C, Wilson M (1992). Spontaneous locomotor hyperactivity in a mouse mutant with a deletion including the Snap gene on chromosome 2. J Neurosci.

[127] Hess E (1996). The use of transgenes and mutations in the mouse to study the genetic basis of locomotor hyperactivity. Methods.

[128] Clayton DF, George JM (1998). The synucleins: a family of proteins involved in synaptic function, plasticity, neurodegeneration and disease. Trends Neurosci.

[129] Polymeropoulos MH, Lavedan C, Leroy E, Ide SE, Dehejia A, Dutra A (1997). Mutation in the alpha-synuclein gene identified in families with Parkinson’s disease. Science.

[130] Goedert M, Grazia Spillantini M, Serpell LC, Berriman J, Smith MJ, Jakes R (2001). From genetics to pathology: tau and a–synuclein assemblies in neurodegenerative diseases. Philos Trans R Soc Lond Ser B: Biol Sci..

[131] Iwai A, Yoshimoto M, Masliah E, Saitoh T (1995). Non-A. beta. Component of Alzheimer’s disease amyloid (NAC) is amyloidogenic. Biochemistry.

[132] Lavedan C (1998). The synuclein family. Genome Res.

[133] Colley WC, Sung T-C, Roll R, Jenco J, Hammond SM, Altshuller Y (1997). Phospholipase D2, a distinct phospholipase D isoform with novel regulatory properties that provokes cytoskeletal reorganization. Curr Biol.

[134] Jenco JM, Rawlingson A, Daniels B, Morris AJ (1998). Regulation of phospholipase D2: selective inhibition of mammalian phospholipase D isoenzymes by α-and β-synucleins. Biochemistry.

[135] Gorbatyuk OS, Li S, Nguyen FN, Manfredsson FP, Kondrikova G, Sullivan LF (2010). α-Synuclein expression in rat substantia nigra suppresses phospholipase D2 toxicity and nigral neurodegeneration. Mol Ther.

[136] Maroteaux L, Scheller RH (1991). The rat brain synucleins; family of proteins transiently associated with neuronal membrane. Brain Res. Mol Brain Res.

[137] Davidson WS, Jonas A, Clayton DF, George JM (1998). Stabilization of alpha-synuclein secondary structure upon binding to synthetic membranes. J Biol Chem.

[138] Murphy DD, Rueter SM, Trojanowski JQ, Lee VM (2000). Synucleins are developmentally expressed, and alpha-synuclein regulates the size of the presynaptic vesicular pool in primary hippocampal neurons. J Neurosci.

[139] Venda LL, Cragg SJ, Buchman VL, Wade-Martins R (2010). α-Synuclein and dopamine at the crossroads of Parkinson’s disease. Trends Neurosci.

[140] Burré J (2015). The synaptic function of α-synuclein. J Parkinson’s Dis.

[141] Tehranian R, Montoya SE, Van Laar AD, Hastings TG, Perez RG (2006). Alpha-synuclein inhibits aromatic amino acid decarboxylase activity in dopaminergic cells. J Neurochem.

[142] Peng X, Tehranian R, Dietrich P, Stefanis L, Perez RG (2005). Alpha-synuclein activation of protein phosphatase 2A reduces tyrosine hydroxylase phosphorylation in dopaminergic cells. J Cell Sci.

[143] Perez RG, Waymire JC, Lin E, Liu JJ, Guo F, Zigmond MJ (2002). A role for α-Synuclein in the regulation of dopamine biosynthesis. J Neurosci.

[144] Abeliovich A, Schmitz Y, Fariñas I, Choi-Lundberg D, Ho WH, Castillo PE (2000). Mice lacking alpha-synuclein display functional deficits in the nigrostriatal dopamine system. Neuron.

[145] Cabin DE, Shimazu K, Murphy D, Cole NB, Gottschalk W, McIlwain KL (2002). Synaptic vesicle depletion correlates with attenuated synaptic responses to prolonged repetitive stimulation in mice lacking α-Synuclein. J Neurosci.

[146] Peña-Oliver Y, Buchman VL, Stephens DN (2010). Lack of involvement of alpha-synuclein in unconditioned anxiety in mice. Behav Brain Res.

[147] Senior SL, Ninkina N, Deacon R, Bannerman D, Buchman VL, Cragg SJ (2008). Increased striatal dopamine release and hyperdopaminergic-like behaviour in mice lacking both alpha-synuclein and gamma-synuclein. Eur J Neurosci.

[148] Mooney MA, McWeeney SK, Faraone SV, Hinney A, Hebebrand J, Nigg JT (2016). Pathway analysis in attention deficit hyperactivity disorder: an ensemble approach. Am J Med Genet B Neuropsychiatr Genet.

[149] Aasen T, Belmonte JCI (2010). Isolation and cultivation of human keratinocytes from skin or plucked hair for the generation of induced pluripotent stem cells. Nat Protoc.

[150] Staerk J, Dawlaty MM, Gao Q, Maetzel D, Hanna J, Sommer CA (2010). Reprogramming of human peripheral blood cells to induced pluripotent stem cells. Cell Stem Cell.

[151] Takahashi K, Tanabe K, Ohnuki M, Narita M, Ichisaka T, Tomoda K (2007). Induction of pluripotent stem cells from adult human fibroblasts by defined factors. Cell.

[152] Re S, Dogan AA, Ben-Shachar D, Berger G, Werling AM, Walitza S (2018). Improved generation of induced pluripotent stem cells from hair derived keratinocytes – a tool to study neurodevelopmental disorders as ADHD. Front Cell Neurosci.

[153] Yde Ohki CM, Grossmann L, Alber E, Dwivedi T, Berger G, Werling AM (2020). The stress-Wnt-signaling axis: a hypothesis for attention-deficit hyperactivity disorder and therapy approaches. Transl Psychiatry.

[154] de la Peña JB, Dela Peña IJ, Custodio RJ, Botanas CJ, Kim HJ, Cheong JH (2018). Exploring the validity of proposed transgenic animal models of Attention-Deficit Hyperactivity Disorder (ADHD). Mol Neurobiol.

[155] Siesser WB, Zhao J, Miller LR, Cheng SY, McDonald MP (2006). Transgenic mice expressing a human mutant beta1 thyroid receptor are hyperactive, impulsive, and inattentive. Genes Brain Behav.

[156] Zhuang X, Oosting RS, Jones SR, Gainetdinov RR, Miller GW, Caron MG (2001). Hyperactivity and impaired response habituation in hyperdopaminergic mice. Proc Natl Acad Sci.

[157] Rubinstein M, Phillips TJ, Bunzow JR, Falzone TL, Dziewczapolski G, Zhang G (1997). Mice Lacking Dopamine D4 Receptors Are Supersensitive to Ethanol, Cocaine, and Methamphetamine. Cell.

[158] Shaywitz BA, Yager RD, Klopper JH (1976). Selective brain dopamine depletion in developing rats: an experimental model of minimal brain dysfunction. Science.

[159] Avale ME, Falzone TL, Gelman DM, Low MJ, Grandy DK, Rubinstein M (2004). The dopamine D4 receptor is essential for hyperactivity and impaired behavioral inhibition in a mouse model of attention deficit/hyperactivity disorder. Mol Psychiatry.

[160] Granon S, Changeux JP (2006). Attention-deficit/hyperactivity disorder: a plausible mouse model?. Acta Paediatr.

[161] Yan TC, Hunt SP, Stanford SC (2009). Behavioural and neurochemical abnormalities in mice lacking functional tachykinin-1 (NK1) receptors: a model of attention deficit hyperactivity disorder. Neuropharmacology.

[162] Sharp SI, McQuillin A, Marks M, Hunt SP, Stanford SC, Lydall GJ (2014). Genetic association of the tachykinin receptor 1 TACR1 gene in bipolar disorder, attention deficit hyperactivity disorder, and the alcohol dependence syndrome. Am J Med Genet Part B: Neuropsychiatr Genet.

[163] Pillidge K, Heal DJ, Stanford SC (2016). The NK1R-/- mouse phenotype suggests that small body size, with a sex- and diet-dependent excess in body mass and fat, are physical biomarkers for a human endophenotype with vulnerability to attention deficit hyperactivity disorder. J Psychopharmacol.

[164] Drerup JM, Hayashi K, Cui H, Mettlach GL, Long MA, Marvin M (2010). Attention-deficit/hyperactivity phenotype in mice lacking the cyclin-dependent kinase 5 cofactor p35. Biol Psychiatry.

[165] Krapacher FA, Mlewski EC, Ferreras S, Pisano V, Paolorossi M, Hansen C (2010). Mice lacking p35 display hyperactivity and paradoxical response to psychostimulants. J Neurochem.

[166] Gong R, Ding C, Hu J, Lu Y, Liu F, Mann E (2011). Role for the membrane receptor guanylyl cyclase-C in attention deficiency and hyperactive behavior. Science.

[167] Huang J, Zhong Z, Wang M, Chen X, Tan Y, Zhang S (2015). Circadian modulation of dopamine levels and dopaminergic neuron development contributes to attention deficiency and hyperactive behavior. J Neurosci.

[168] D’Andrea I, Fardella V, Fardella S, Pallante F, Ghigo A, Iacobucci R (2015). Lack of kinase-independent activity of PI3Kγ in locus coeruleus induces ADHD symptoms through increased CREB signaling. EMBO Mol Med.

[169] Zhou M, Rebholz H, Brocia C, Warner-Schmidt JL, Fienberg AA, Nairn AC (2010). Forebrain overexpression of CK1delta leads to down-regulation of dopamine receptors and altered locomotor activity reminiscent of ADHD. Proc Natl Acad Sci USA.

[170] Yang P, Cai G, Cai Y, Fei J, Liu G (2013). Gamma aminobutyric acid transporter subtype 1 gene knockout mice: a new model for attention deficit/hyperactivity disorder. Acta Biochim et Biophys Sin.

[171] Chen L, Yang X, Zhou X, Wang C, Gong X, Chen B (2015). Hyperactivity and impaired attention in Gamma aminobutyric acid transporter subtype 1 gene knockout mice. Acta Neuropsychiatr.

[172] Granon S, Faure P, Changeux J-P (2003). Executive and social behaviors under nicotinic receptor regulation. Proc Natl Acad Sci.

[173] Guillem K, Bloem B, Poorthuis RB, Loos M, Smit AB, Maskos U (2011). Nicotinic acetylcholine receptor β2 subunits in the medial prefrontal cortex control attention. Science.

[174] Zimmermann A-M, Jene T, Wolf M, Görlich A, Gurniak CB, Sassoè-Pognetto M (2015). Attention-deficit/hyperactivity disorder–like phenotype in a mouse model with impaired actin dynamics. Biol Psychiatry.

[175] Won H, Mah W, Kim E, Kim JW, Hahm EK, Kim MH (2011). GIT1 is associated with ADHD in humans and ADHD-like behaviors in mice. Nat Med.

[176] Kakefuda K, Oyagi A, Ishisaka M, Tsuruma K, Shimazawa M, Yokota K (2010). Diacylglycerol Kinase β knockout mice exhibit lithium-sensitive behavioral abnormalities. PLOS ONE.

[177] Ishisaka M, Kakefuda K, Oyagi A, Ono Y, Tsuruma K, Shimazawa M (2012). Diacylglycerol kinase β knockout mice exhibit attention-deficit behavior and an abnormal response on methylphenidate-induced hyperactivity. PLoS One.

[178] Xie K, Ge S, Collins VE, Haynes CL, Renner KJ, Meisel RL (2012). Gβ5-RGS complexes are gatekeepers of hyperactivity involved in control of multiple neurotransmitter systems. Psychopharmacology.

[179] Moon J-s, Beaudin A, Verosky S, Driscoll L, Weiskopf M, Levitsky D (2006). Attentional dysfunction, impulsivity, and resistance to change in a mouse model of fragile X syndrome. Behav Neurosci.

[180] Kramvis I, Mansvelder HD, Loos M, Meredith R (2013). Hyperactivity, perseveration and increased responding during attentional rule acquisition in the Fragile X mouse model. Front Behav Neurosci.

[181] Chaudhry A, Noor A, Degagne B, Baker K, Bok L (2015). Brady mF, et al. Phenotypic spectrum associated with PTCHD1 deletions and truncating mutations includes intellectual disability and autism spectrum disorder. Clin Genet.

[182] Wells MF, Wimmer RD, Schmitt LI, Feng G, Halassa MM (2016). Thalamic reticular impairment underlies attention deficit in Ptchd1 Y/− mice. Nature.

[183] Gao Y, Heldt SA (2015). Lack of neuronal nitric oxide synthase results in attention deficit hyperactivity disorder-like behaviors in mice. Behav Neurosci.

[184] Gerber DJ, Sotnikova TD, Gainetdinov RR, Huang SY, Caron MG, Tonegawa S (2001). Hyperactivity, elevated dopaminergic transmission, and response to amphetamine in M1 muscarinic acetylcholine receptor-deficient mice. Proc Natl Acad Sci USA.

[185] Miyakawa T, Yamada M, Duttaroy A, Wess J (2001). Hyperactivity and intact hippocampus-dependent learning in mice lacking the M1 muscarinic acetylcholine receptor. J Neurosci.

[186] Kobayashi M, Nakatani T, Koda T, Matsumoto K-i, Ozaki R, Mochida N (2014). Absence of BRINP1 in mice causes increase of hippocampal neurogenesis and behavioral alterations relevant to human psychiatric disorders. Mol Brain.

[187] Berkowicz SR, Featherby TJ, Whisstock JC, Bird PI (2016). Mice Lacking Brinp2 or Brinp3, or both, exhibit behaviors consistent with neurodevelopmental disorders. Front Behav Neurosci.

[188] Rivero O, Selten MM, Sich S, Popp S, Bacmeister L, Amendola E (2015). Cadherin-13, a risk gene for ADHD and comorbid disorders, impacts GABAergic function in hippocampus and cognition. Transl Psychiatry.

[189] Drgonova J, Walther D, Hartstein GL, Bukhari MO, Baumann MH, Katz J (2016). Cadherin 13: human cis-regulation and selectively-altered addiction phenotypes and cerebral cortical dopamine in knockout mice. Mol Med.

[190] Napolitano F, Bonito-Oliva A, Federici M, Carta M, Errico F, Magara S (2010). Role of aberrant striatal dopamine D1 receptor/cAMP/protein kinase A/DARPP32 signaling in the paradoxical calming effect of amphetamine. J Neurosci.

[191] Salahpour A, Ramsey AJ, Medvedev IO, Kile B, Sotnikova TD, Holmstrand E (2008). Increased amphetamine-induced hyperactivity and reward in mice overexpressing the dopamine transporter. Proc Natl Acad Sci.

[192] Viggiano D, Vallone D, Welzl H, Sadile AG (2002). The Naples high- and low-excitability rats: selective breeding, behavioral profile, morphometry, and molecular biology of the mesocortical dopamine system. Behav Genet.

[193] Sadile AG, Lamberti C, Siegfried B, Welzl H (1993). Circadian activity, nociceptive thresholds, nigrostriatal and mesolimbic dopaminergic activity in the Naples High-and Low-Excitability rat lines. Behav Brain Res.

[194] Magara F, Ricceri L, Wolfer DP, Lipp HP (2000). The acallosal mouse strain I/LnJ: a putative model of ADHD?. Neurosci Biobehav Rev.

[195] dela Peña IJI, Botanas CJ, de la Peña JB, Custodio RJ, dela Peña I, Ryoo ZY (2019). The Atxn7-overexpressing mice showed hyperactivity and impulsivity which were ameliorated by atomoxetine treatment: a possible animal model of the hyperactive-impulsive phenotype of ADHD. Prog Neuro-Psychopharmacol Biol Psychiatry.

[196] Pennanen L, van der Hart M, Yu L, Tecott LH (2013). Impact of Serotonin (5-HT)2C receptors on executive control processes. Neuropsychopharmacology.

[197] Papaleo F, Erickson L, Liu G, Chen J, Weinberger DR (2012). Effects of sex and COMT genotype on environmentally modulated cognitive control in mice. Proc Natl Acad Sci.

[198] Cui Y, Costa RM, Murphy GG, Elgersma Y, Zhu Y, Gutmann DH (2008). Neurofibromin regulation of ERK signaling modulates GABA release and learning. Cell.

[199] Loos M, Mueller T, Gouwenberg Y, Wijnands R, van der Loo RJ, Birchmeier C (2014). Neuregulin-3 in the mouse medial prefrontal cortex regulates impulsive action. Biol Psychiatry.

